# Mechanochemical Synthesis of Bi_2_VO_5.5_ for Improved Photocatalytic Dye Degradation

**DOI:** 10.1002/gch2.202200172

**Published:** 2023-01-17

**Authors:** Manish Kumar, Rahul Vaish, Tae Hyun Sung, Anuruddh Kumar, El Sayed Yousef

**Affiliations:** ^1^ School of Mechanical and Materials Engineering Indian Institute of Technology Mandi Mandi 175005 India; ^2^ Department of Electrical Engineering Hanyang University 222, Wangsimni‐ro, Seongdong‐gu Seoul 04763 Korea; ^3^ Center for Creative Convergence Education, Hanyang University 222, Wangsimni‐ro, Seongdong‐gu 04763 Seoul Korea; ^4^ Research Center for Advanced Materials Science (RCAMS) King Khalid University 61413, P. O. Box 9004 Abha 9004 Saudi Arabia; ^5^ Physics Departement Faculty of Science King Khalid University P. O. Box 9004 Abha 9004 Saudi Arabia

**Keywords:** ball‐milled, Bi _2_VO _5.5_, germination index, photocatalysis

## Abstract

A single‐phase Bi_2_VO_5.5_ powder is formed effectively through a mechanochemical ball milling approach at 650 °C in 5 h and its photocatalytic performance on methylene blue dye is explored. X‐ray diffraction and Raman spectroscopy analytical instruments are utilized to confirm the phase formation. The evident presence of irregular‐shaped grains is affirmed using a scanning electron microscope. To ascertain the chemical condition of the components present, the Bi_2_VO_5.5_ powdered sample undergo an X‐Ray photoelectron spectroscopy investigation. The sample is analyzed using a time‐dependent photocurrent to discern its charge carrier transportation behavior. A photocatalytic study using Bi_2_VO_5.5_ powder produced through the mechanochemical ball milling method has not been explored till now. The efficacy of the ball‐milled Bi_2_VO_5.5_ powder to attain enhanced photocatalytic efficiency which hasn't been investigated till now, is explored. The ball‐milled Bi_2_VO_5.5_ sample achieved 70% degradation efficiency when performing the photocatalysis investigation. The photocatalytic dye degradation discerns pseudo‐first‐order kinetics and achieves a notable *k* value of 0.00636 min^−1^. The scavenger test indicates that h^+^ radicals are the prominent active species during the photocatalysis experiment. The germination index is determined by conducting a phytotoxicity test with the use of *Vigna radiata* seeds. Here ball‐milled Bi_2_VO_5.5_ powder attains enhanced dye degradation efficiency.

## Graphical abstract

The single‐phase Bi_2_VO_5.5_ powder is formed effectively through a mechanochemical ball milling approach at 650 °C in 5 h. The ball‐milled Bi_2_VO_5.5_ sample achieves 70% degradation efficiency when performing the photocatalysis investigation. The scavenger test indicates that h^+^ radicals are the prominent active species during the photocatalysis experiment.

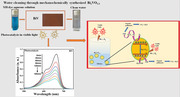

## 1 Introduction

Rapid industrial development has led to increased water pollution around the world. Textile industries discharge colored and toxic dye effluents which contaminate both surface and groundwater.^[^ ^1^, ^2^ ^]^ At the present time efficient, eco‐friendly, and green energy sources to eradicate environmental pollution are of great interest to researchers. Photocatalysis is a green and effective method to eradicate organic pollutants from water bodies since it can effectively degrade organic pollutants into much smaller molecules like H_2_O, CO_2_, etc.^[^ ^3^ ^]^ Dye degradation using photocatalysis has been given due consideration due to its advantageous features like non‐toxicity, low cost, reduced secondary pollutants, and low concentration contaminant oxidation feasible at room temperature.^[^ ^4^, ^5^ ^]^ Anatase TiO_2_ is used as a commercialized photocatalyst at the present owing to its low price, chemical stability, high oxidizing power, and non‐toxicity.^[^ ^6^, ^7^, ^8^ ^]^ TiO_2_ possesses a wide bandgap (3.20 eV) which confines its functioning only in the UV region. Also, the lifetime of the photo‐induced charge carriers is relatively short which gives low catalytic efficiency. These factors confine the usage of TiO_2_ as a photocatalyst and further urge the researchers to develop a better photocatalyst that would be visible light active.^[^ ^6^, ^8^ ^]^

Recently great insistence is given to developing visible light active Bi‐based photocatalysts due to low‐cost raw materials and their apparent electronic structure.^[^ ^9^ ^]^ BiVO_4_, Bi_2_O_3_, Bi_2_WO_6_, CaBi_2_O_4_, BiNbO_4_, Bi_2_VO_5.5_, Bi_2_MoO_6_, etc. are a few recently proclaimed bismuth‐based photocatalysts.^[^ ^10^, ^11^ ^]^ The oxides containing Bi^3+^ show good photocatalytic properties owing to the existence of hybridized O (2p) as well as Bi (6s) valence bands.^[^ ^11^ ^]^ Bi_2_WO_6_, Bi_2_MoO_6_, and Bi_2_VO_5.5_ possess layered structure which greatly assists in charge carrier transportation and thus demonstrate excellent catalytic activity over wide visible light response.^[^ ^1^ ^]^

Bi_2_VO_5.5_ (BiV) is a photocatalyst that shows ferroelectric properties below 725 K.^[^ ^12^, ^13^, ^14^ ^]^ It is utilized in a variety of applications like electrode materials for lithium‐ion batteries, solid‐state electrolytes, and gas sensors.^[^ ^15^ ^]^ BiV can be synthesized through various approaches like co‐precipitation, solid‐state reaction, microwave, and sol‐gel.^[^ ^12^, ^13^, ^16^ ^]^ It is evident that catalytic dye degradation efficiency counts on powder surface area as well as particle size. Most methods of synthesizing BiV produce a small surface area with a large particle size owing to the lower solubility of BiV in an aqueous solution or high calcination temperature.^[^ ^17^, ^18^ ^]^ An effort has been laid to synthesize nano‐sized BiV to attain high photocatalytic efficiency. Kundapura et. al reported the synthesis of nano‐sized BiV.^[^ ^19^ ^]^ To attain high degradation efficiency, charge carrier recombination needs to be further improved.^[^ ^20^ ^]^ High energy‐induced ball milling is considered an optimum approach to attain reduced crystalline domain size.^[^ ^21^ ^]^

BiV is from the Aurivillius family member that possesses the generalized formula (Bi_2_O_2_)^2+^ (A_n‐1_B_n_O_3n+1_)^2−^, where B symbolizes hexa‐, tetra‐, and pentavalent ions, *n* symbolizes counts of perovskite blocks crammed between Bi_2_O_2_ layers and A symbolize di‐, tri‐, and mono‐valent ions.^[^ ^14^ ^]^ BiV possesses a layered structure similar to BiVO_4_.^[^ ^22^ ^]^ BiV has a low bandgap which acclaims its usage over a large visible light absorption range.^[^ ^22^ ^]^ Xie. et. al used Au nanoparticles loaded on Bi_2_VO_5.5_ to degrade methylene blue (MB) achieving 85.2% efficiency.^[^ ^23^ ^]^ Xie. et. al reported attaining 89.97% MB dye disintegration efficiency in presence of simulated sunlight with Bi_2_VO_5.5_/Bi_2_O_3_ composite films.^[^ ^1^ ^]^ Jianmin wang et. al. reported attaining 95% methylene orange (MO) disintegration performance in visible light with BiVO_4_/ Bi_2_VO_5.5_ nanostructure.^[^ ^22^ ^]^

Recently we reported 60% MB dye degradation efficiency using a ball‐milled BiVO_4_ powdered sample.^[^ ^24^ ^]^ Phamalee et. al reported photocatalytic MB dye degradation of Bi_2_VO_5.5_ powder prepared through the microwave method.^[^ ^12^ ^]^ Mechanochemical ball milling (MBM) is a unique method to yield reduced‐sized particles. The mechanochemical approach comprises pulverizing and chemical reactions during vigorous milling as a result of high‐energy collisions between the reactant particles and the grinding media.^[^ ^25^ ^]^ High‐energy ball milling with a mechanochemical approach has recently drawn a lot of interest as a cheap, solvent‐free, and low‐temperature synthesis technique.^[^ ^26^, ^27^ ^]^ This procedure is environmentally benign because it produces no hazardous or chemical waste and does not require sophisticated machinery or vacuum facilities.^[^ ^28^ ^]^ High‐energy milling may result in chemical reactions owing to the extreme impact and friction between the jar walls and the balls. Thus, the reactions are possible at considerably lower temperatures and in the solid state.^[^ ^28^ ^]^ The mechanochemical reactions that take place at room temperature and atmospheric pressure have benefits including reproducibility, high yield, ease of operation, and scalability.^[^ ^29^, ^30^ ^]^ The efficiency of mechanochemical processes depends on a variety of factors, including mill rotation speed, duration, temperature, and milling environment.^[^ ^31^ ^]^ It is vital to create a unique technique to achieve the efficient production of Bi_2_VO_5.5_ with cost effectiveness, environmental friendliness, and controllability in order to comply with ever‐stricter environmental rules. A photocatalytic study using Bi_2_VO_5.5_ powder produced through the MBM method has not been explored till now. In this work, we explored the ball‐milled Bi_2_VO_5.5_ (BiV) which belongs to the Bi–V–O system same as BiVO_4_. BiV is prepared with a specific rotation speed per minute (rpm) of the synthesis chamber in addition to the ball‐to‐powder ratio. The decrease in the particle size during the milling process instigates defects and micro‐stress given the meta‐stable high energy state. So, in order to relinquish the defects induced, BiV is put through post‐annealing treatment.^[^ ^24^, ^32^, ^33^ ^]^ Here, we have tried to explore the efficacy of the ball‐milled BiV powder to attain enhanced photocatalytic efficiency which has not been investigated till now. The ball‐milled powder will surely enhance dye degradation efficiency in less time when used at the industrial level for wastewater treatment.

## 2 Experimental Section

### 2.1 Synthesis of Bi_2_VO_5.5_ Powder

**Scheme 1:**
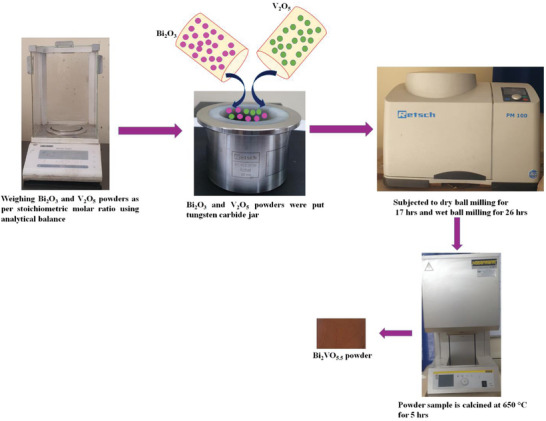
The synthesis method of BiV powder.

The mechanochemical ball milling (MBM) process was an efficient approach to producing submicron and nano‐sized materials. Repeated fracturing and welding assure solid‐state reaction occurrence of each indulged material caused the final synthesis of powder material. This method attained crystalline order submicron‐sized powdered material of proper stoichiometry that eventually alloyed the present chemical elements. The BiV powder synthesized through the MBM method is shown in **Scheme** **1**. V_2_O_5_ and Bi_2_O_3_ oxide powders in conformity to the stoichiometric molar ratio were put in a tungsten carbide jar with a capacity of 250 ml. 10 pieces of tungsten carbide balls each of 20 mm diameter were used during the milling process. Retsch planetary ball mill (PM100) setup was utilized for room temperature milling process for 17 h at 300 rpm. To cool the system, a halt of 5 min was given after every elapse of 30 min. The wet milling process at 300 rpm for 26 h was further induced to attain reduced particle size with a specific weight ratio of BiV powder: yttria‐stabilized zirconia (YSZ) balls: ethanol ratio = 1:20:0.5. The obtained powders after separating YSZ balls were placed for annealing at 650 °C for 5 h so as to eradicate the induced defects crept during the continuous ball milling. Post‐annealing we get the ball‐milled Bi_2_VO_5.5_ (BiV) powders. In view to demonstrate comparison in photocatalytic activity, a distinct, not a ball‐milled Bi_2_VO_5.5_ (NBM BiV) sample was synthesized using V_2_O_5_ and Bi_2_O_3_ oxide powders in proper stoichiometric molar ratio. The powders were ground using a mortar pestle and then later subjected to a calcination temperature of 750 °C for 8 h in view to affirm the Bi_2_VO_5.5_ phase. The NBM BiV formed was validated through X‐ray diffraction. Further, a distinct not ball‐milled Bi_2_VO_5.5_ (BiV‐650) was synthesized by collecting V_2_O_5_ and Bi_2_O_3_ oxide powders gathered in proper stoichiometric molar ratio. The powders were ground using a mortar pestle and then later heated to a calcination temperature of 650 °C for 5 h.

### 2.2 Characterization of Bi_2_VO_5.5_

X‐ray diffraction (XRD, Rigaku diffractometer, made in Japan, 9 kW, equipped with Cu‐K*a* rotating anode) instrument was utilized to affirm phase formation for the samples BiV, BiV‐650, and NBM BiV. The powdered sample was analyzed at a scan rate of 2° min^−1^ throughout the 2*a* angle range from 10–60°. HORIBA (LabRAM HR Evolution, made in Japan) spectrometer was utilized to analyze the bonding as well as the structure in the BiV sample. For Raman spectra acquisition, a laser excitation wavelength of 532 nm with 600 gratings and 10% power was used to examine the specimen between 350–1100 cm^−1^ range. A scanning electron microscope (FE‐SEM, Nova Nano SEM‐450) was employed to understand the surface morphology as well as the microstructures. Energy dispersive spectroscopy (EDS) inbuilt into SEM provided the dissemination of the compositional element present in the BiV powder. UV–vis spectrophotometer (SHIMADZU) instrument was taken in use to acquire the intensity of the absorbance peak.

### 2.3 Photocurrent Measurement

Current‐time curves of the BiV sample were collected using an electrochemical workstation (Metrohm Autolab B.V, AUT86543). Three electrodes arrangement was used where Ag‐AgCl wire was utilized as the reference and the working electrode while the platinum wire acted as the counter electrode. Visible light was offered through two bulbs each of 15 W from Havells company. A few cycles of ON as well as OFF of the visible light source were done to acquire the photocurrent response. The electrolyte prepared was 0.1 M phosphate‐buffered saline solution. In order to develop the working electrode, 5 mg catalyst, and 20 µL Nafion solution were appended to 1 mL ethanol. This prepared catalyst ink was well dispersed by subjecting it to ultrasonication for 30 min post which the cleansed glassy carbon electrode was surface coated with ≈10 µL of catalyst ink. Appropriate drying of the coat on the electrode surface was affirmed prior to using it as the working electrode.

### 2.4 Analysis of the Bandgap

Diffuse reflectance spectroscopy (DRS) was utilized to determine the bandgap of the synthesized samples. To obtain the direct bandgap, the acquired absorption spectra collected from DRS were transformed into Tauc's plot (drawing a plot between E vs (*aE*)^2^) as suggested in numerous literary works.^[^ ^34^, ^35^ ^]^

### 2.5 Analysis of Photocatalytic Performance

The photocatalytic assessment of the BiV powdered sample was estimated through the disintegration of MB dye solution in the visible light illumination. In the photocatalysis experiment, 0.2 g of the powdered sample was used. Before starting the photocatalytic evaluation, the dye's adsorption–desorption saturation was precisely attained. As the adsorption equilibrium is acquired, the used dye was replaced with a fresh 10 ml dye with a concentration of 5 mg L^−1^ which is considered the primary starting dye. The BiV powdered sample was immersed in the dye solution and positioned under two light bulbs (15 W each from Havells company) emitting the visible light illumination. The approaching light source was positioned 12 cm above the sample. An uninterrupted stirring at a 500 rpm rate was subjected to the sample throughout the entire experiment. The photocatalytic activities were evaluated through the adsorption peak intensity. For this, the test sample was acquired after every regular interval and after evaluating the absorbance, the test sample was cautiously added back into the beaker to ensure a consistent volume throughout. The MB dye's degradation rate percentage was calculated as per Equation (1).^[^ ^36^, ^37^ ^]^

(1) $$\begin{array}{c} \%\;\text{removal}\;\text{of}\;\text{MB}\;\text{dye}\;=\;\frac{C_{o}-C}{C_{o}}\;\times \;100 \end{array}$$

where the symbols *C_o_* and *C* illustrate the previous starting MB dye concentration value and their concentration after the passing of “*t*” time, respectively.

## 3 Results and Discussion

**Figure 1:**
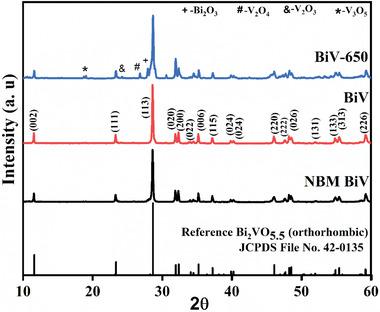
XRD results of the prepared BiV, BiV‐650, and NBM BiV powders.

**Figure** **1** exhibits the XRD patterns of the synthesized BiV, NBM BiV, and BiV‐650 powdered samples. The acquired XRD patterns of BiV and NBM BiV convey that all the diffracted peaks acquired are in conformance with the specific JCPDS reference file of the orthorhombic BiVO_5.5_ (File No. 42–0135). As can be viewed that there is single phase formation for BiV and NBM BiV without the occurrence of any secondary peak. In context of BiV‐650, we find the presence of secondary peak formation of V_3_O_5_, V_2_O_3_, V_2_O_4,_ and Bi_2_O_3_ at 18.8°, 24.2°, 26.8°, and 27.9° respectively apart from Bi_2_VO_5.5_ phase. MBM method produced fine particles which assisted in synthesizing Bi_2_VO_5.5_ single phase viable at 650 °C within 5 h, so this work produces Bi_2_VO_5.5_ single phase at lessening synthesis time than the reported 24 h annealing at 1020 K.^[^ ^38^ ^]^ The powder through the mortar pestle grinding method could not be synthesized at 650 °C within 5 h giving an additional phase, so we intend to discard the BiV‐650 powdered sample in our further experimental investigations.

**Figure 2:**
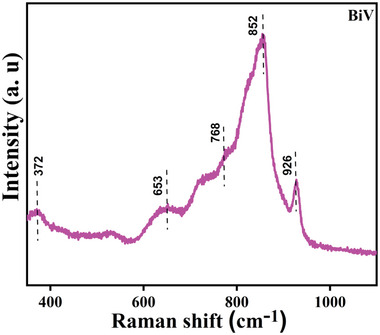
The Raman spectrum of BiV powder.

**Figure** **2** exhibits the Raman bands correlated to the synthesized BiV sample. The Raman spectra were analyzed over the Raman shift range of 350–1100 cm^−1^. The acquired Raman bands were observed at 926, 852, 768, 653, and 372 cm^−1^. These procured bands are in conformance with the previous literary works.^[^ ^39^, ^40^ ^]^ The band procured at 372 cm^−1^ denotes vibrational mode symmetric bending of the V–O bonds while the procured bands at 768 and 653 cm^−1^ denote double coordinated (V–O–V) oxygen atom.^[^ ^39^ ^]^ The band procured at 852 cm^−1^ denotes vibrational short‐range V–O bonds while the weak mode present at 925 cm^−1^ denotes V^4+^=O unit.^[^ ^40^ ^]^ Vanadium when existing in a mixed valence state of +5 and +4, the presence of a weak vibrational mode signifies the signature of the V^4+^=O.^[^ ^40^ ^]^

**Figure 3:**
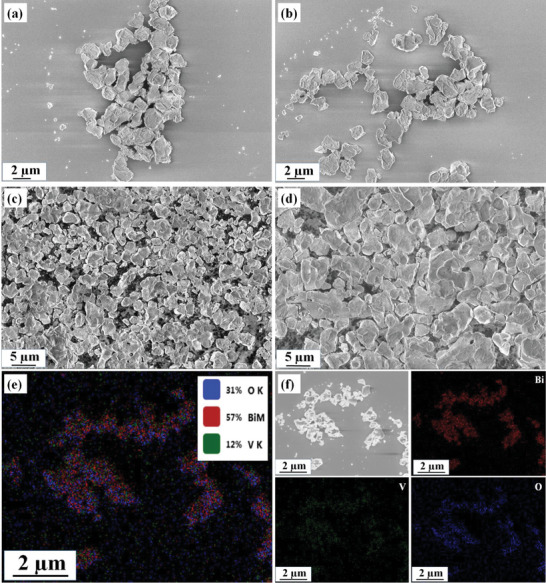
The SEM images of a–c) BiV sample, d) NBM BiV, and e,f) EDS elemental color mapping analysis of BiV.

**Figure** **3** displays the sample's surface morphology using SEM micrographs. The evident existence of the irregular‐shaped grains of the BiV sample is evidenced in Figure 3a–c. Figure 3d displays the irregularly shaped morphology of the NBM BiV sample. Using the ball milling technique, we have successfully reduced the particle size in the submicron range evidenced by the SEM micrographs. We can evidence the difference in the particle size between the ball‐milled and not‐ball sample of Bi_2_VO_5.5_ from Figures 3c,d. For proper phase identification, EDS elemental color mapping for the BiV sample was conducted. Figure 3c,d presents the selected area of the mapping along with the confirmation of the existing Bi, V, and O elements.

**Figure 4:**
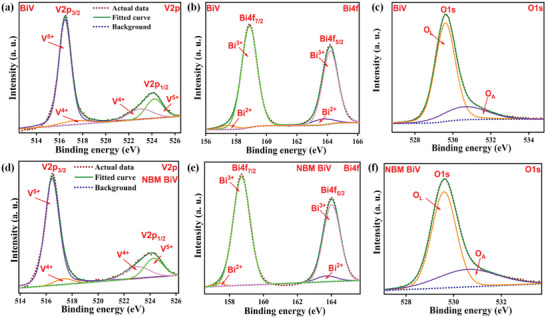
The XPS for V2p, Bi4f, and O1s of a–c) BiV and d–f) NBM BiV powder sample.

To ascertain the chemical condition of the components present, the BiV and NBM BiV powdered samples underwent an XPS investigation. **Figures** **4**a–f depict the V2p, Bi4f, and O1s XPS spectra of BiV and NBM BiV respectively. For both BiV and NBM BiV samples, the V2p spectrum's V2p_3/2_ and V2p_1/2_ components were first segmented into corresponding V^4+^ and V^5+^ components. While peaks at 517.3 and 523 eV are in conformity with the V^4+^ oxidation state, peaks at 524.2 and 516.4 eV are congruent with the V^5+^ oxidation state.^[^ ^41^ ^]^ Asymmetric O1s spectra were segmented into O_L_ and O_A_ major components. Here, the symbols O_L_ and O_A_ stand for the lattice oxygen (O^2−^ oxidation state) and the presence of oxygen vacancies, respectively.^[^ ^42^ ^]^ The inherent defects that seeped into the material during processing through heat treatment are what lead to localized oxygen vacancies. For both BiV and NBM BiV samples, the asymmetric Bi4f_7/2_ and Bi4f_5/2_ segments of the Bi4f spectrum were initially segregated into corresponding Bi^2+^ and Bi^3+^ parts. For the BiV sample, the Bi^2+^ oxidation state is compatible with the peaks at 163.7 and 157.3 eV, whereas the Bi^3+^ oxidation state is significant with the peaks at 158.8 and 164.1 eV. For the NBM BiV sample, the Bi^2+^ oxidation state is compatible with 163.7 and 157.3 eV as the optimum values, whereas the Bi^3+^ oxidation state is significant with 164 and 158.7 eV as the optimum values.^[^ ^42^, ^43^ ^]^ We notice a feeble variation in the presence of peaks for the Bi^3+^ oxidation state in the context of the BiV and NBM BiV samples. This may be due to intrinsic defects introduced during processing through heat treatment. Due to the extra charge being bound in certain electron pair configurations surrounding the Bi and V atoms as well as at the vacancy area, Bi^3+^ and V^5+^ are reduced to Bi^2+^ and V^4+^, respectively. In addition to the already present Bi^3+^ and V^5+^, we thereby detect the existence of Bi^2+^ and V^4+^ in the BiV and NBM BiV samples.^[^ ^44^ ^]^ Thus, we could infer that the efficient performance difference in the photocatalytic dye degradation is due to the ball milling‐induced particle size reduction.

**Figure 5:**
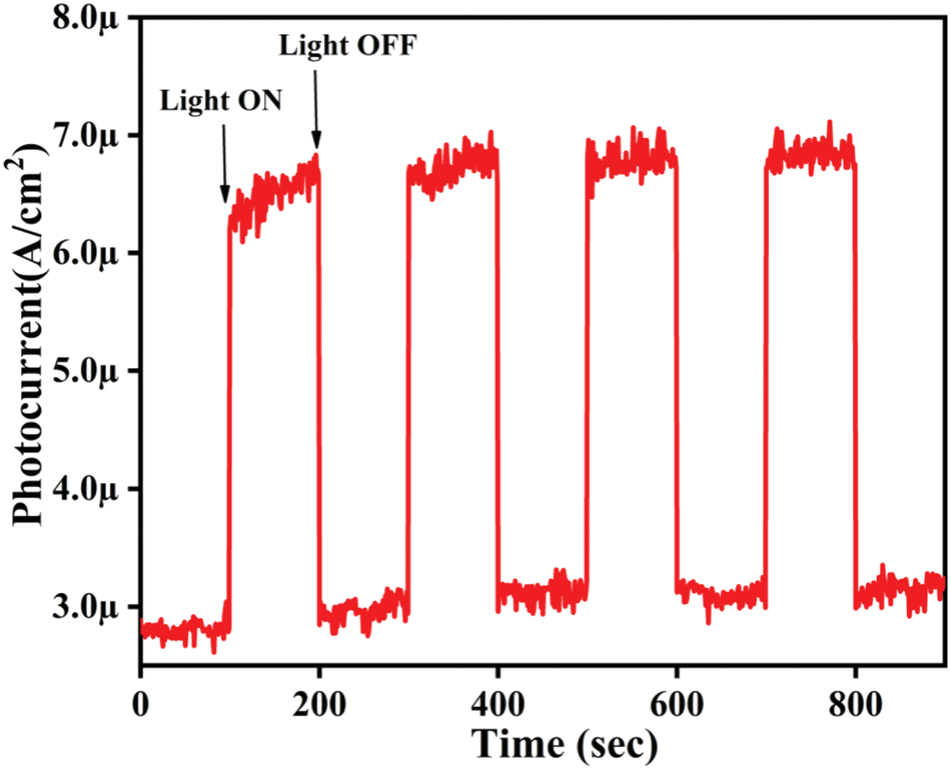
Photocurrent versus time plot for BiV powder.

**Figure** **5** displays the time‐dependent photocurrent evaluation of the BiV powdered sample to discern its charge carrier transportation behavior. BiV achieves a less dark current density (≈2.8 µA cm^−2^) prior to dye degradation and upon the irradiation of the visible light, it showed an enhanced photocurrent density of ≈6.5 µA cm^−2^. As a result, we attained a visible light to the dark current ratio of nearly 2.3 times. The attainment of a high value of photocurrent density supports the efficacious separation of photogenerated charge carriers. The replication of the photocurrent density value for every irradiation supports the fact that the BiV material's photocurrent response is reversible.^[^ ^45^, ^46^ ^]^

**Figure 6:**
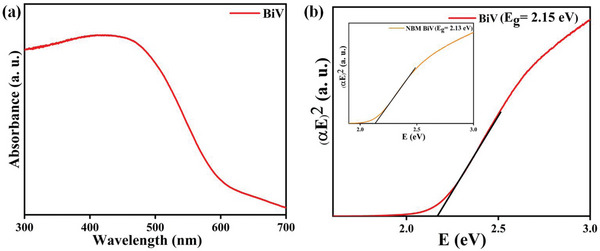
a) BiV powdered sample's absorbance spectrum and b) the bandgap evaluation using Tauc's plot of BiV (inset shows bandgap of NBM BiV).

**Figure** **6**a displays the acquired DRS spectrum of the BiV sample. It displays the acquired optical absorption edge of the ball‐milled BiV sample. Figure 6b displays the investigated bandgap of the BiV sample as 2.15 eV. The bandgap of NBM BiV (2.13 eV) is included in the inset of Figure 6b. It's evident that as explored bandgap of BiV and NBM BiV being almost similar lies in the visible range, and thus the photocatalysis experiment can be assessed in the visible light for the samples.

**Figure 7:**
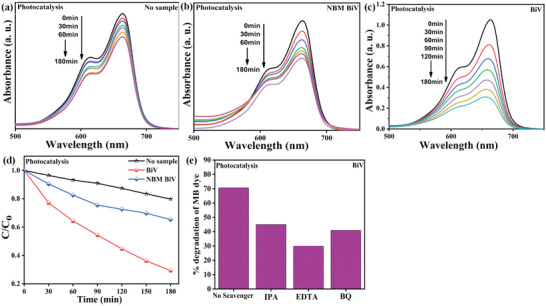
Absorption spectra obtained during photocatalytic experiment utilizing a) No sample, b) NBM BiV, and c) BiV powdered sample. d) $\frac{C}{C_{o}}$versus time graph for the photocatalytic experiment with and without using the sample. e) Influence of distinct scavengers on the photocatalytic dye disintegration using BiV sample.

The photocatalysis experiment for no sample, NBM BiV, and BiV powder was evaluated by degrading MB dye and the collected results have been displayed in **Figure** **7**a–c. Before starting the photocatalytic assessment, the adsorption‐desorption saturation concerning the dye solution was precisely reached. Photocatalytic assessment in visible light irradiation was evaluated by noting the decreased UV–vis MB dye's absorption peak spectra with increasing visible light irradiation time duration as shown in Figure 7a–c. The pollutant dye solution is decolorized is evidenced by the steady decrease in peak intensity with increased time. Figure 7d displays the $\frac{C}{C_{o}}$ versus time plots attained with and devoid of BiV sample use while photocatalysis assessment. Without a sample, the control dye attained 20% MB degradation in 180 min during visible light illumination due to the photolysis process. BiV and NBM BiV samples attained 70% and 35% MB degradation efficiency in 180 min during the visible light illumination respectively. BiV and NBM BiV samples achieved 50% and 15% improvement in the dye degradation efficiency respectively concerning that acquired by the control sample. The difference in the photocatalytic efficiency attained by BiV and NBM BiV samples is due to the difference in the particle size of both the sample which has been demonstrated through the SEM micrographs as well. We evidenced that ball milling enhanced the catalytic performance, as a 50% improvement in the degradation efficiency is achieved by BiV concerning the NBM BiV. Scavengers such as ethylenediaminetetraacetic acid (EDTA), p‐benzoquinone (p‐BQ), and isopropanol (IPA) were appended separately to the MB dye solution containing the BiV sample during photocatalysis in view to seize the active species like holes (h^+^), superoxide radical (•$O_{2}^{-}$), and hydroxyl radical (•OH) respectively.^[^ ^47^, ^48^, ^49^ ^]^ Figure 7e displays that the EDTA scavenger which scavenges h^+^ has greatly impacted the photocatalytic efficiency. Thus, the scavenger test proclaims h^+^ radical as the prominent active species during the photocatalysis experiment. The evaluation of photocatalytic dye degradation utilizing various catalysts synthesized mechanochemically is shown in **Table** **1**.

**Table 1:** Photocatalytic dye degradation utilizing various catalysts synthesized mechanochemically

Catalyst (Powder form)	Model dye	Energy source	Starting concentration (volume usage)	Analysis Time [min]	Dye degradation [%]
Cu_2_ZnSnS_4_^[^ ^26^ ^]^	Methylene Blue (MB)	Visible light	5 mg L^−1^ (‐)	100	100%
0.5CdS/Mg‐Al LDH‐precursor^[^ ^50^ ^]^	MB	Xenon lamp	10 mg L^−1^ (250 mL)	90	94.7%
Bi_2_S_3_/Zn‐Al LDH^[^ ^51^ ^]^	MB	Xenon lamp	5 mg L^−1^ (250 mL)	120	90.1%
Bi_2_VO_5.5_ (This study)	MB	Visible light	5 mg L^−1^ (10 mL)	180	70%
WO_3_(5.0 wt.%)/g‐C_3_N_4_^[^ ^52^ ^]^	MB	Xenon lamp	0.9 × 10^−5^ mol L^−1^ (300 mL)	120	98.5%
S‐doped TiO_2_^[^ ^53^ ^]^	MB	Visible light	0.02 mM (100 mL)	90	99%
BiVO_4_^[^ ^24^ ^]^	MB	Visible light	5 mg L^−1^ (10 mL)	240	60%
CoFe_2_O_4_−RGO^[^ ^54^ ^]^	MB	Xenon lamp	20 mg L^−1^ (40 mL)	180	93.1%
Ta_2_O_5_ nanoparticles^[^ ^55^ ^]^	MB	Hg lamp	10 mg L^−1^ (50 mL)	180	81%

**Figure 8:**
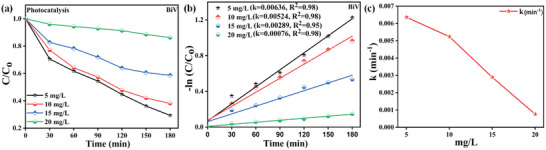
a) $\frac{C}{C_{o}}$ versus time graph acquired using BiV sample in distinct dye concentrations for photocatalysis, b) graph between −ln$\left(\frac{C}{C_{o}}\right)$ versus time in a distinct concentration of dye for photocatalysis experiment and c) graph between the kinetic rate constant “*k*” versus distinct concentration of MB dye (mg L^−1^).

**Figure** **8**a illustrates the $\frac{C}{C_{o}}$ versus time graph procured with BiV sample in distinct dye concentrations (5, 10, 15, and 20 mg L^−1^) during the photocatalysis experiment. Figure 8b displays the −ln$\left(\frac{C}{C_{o}}\right)$ versus time graph obtained with the BiV sample in distinct concentrations of dye during the photocatalysis experiment. The photocatalytic dye disintegration discerns the pseudo‐first‐order kinetic following Equation (2).^[^ ^56^, ^57^ ^]^

(2) $$\begin{array}{c} \ln\frac{C}{C_{o}}=-kt \end{array}$$

where the “*k*” symbol signifies the kinetic rate constant evaluated by the slope of the $\ln\frac{C}{C_{o}}$ versus time “*t*” linear plot. Kinetic rate constants of 0.00636, 0.00524, 0.00289, and 0.00076 min^−1^ were achieved with the distinct dye concentrations of 5, 10, 15, and 20 mg L^−1^ respectively. We find that a dye concentration of 5 mg L^−1^ achieves the maximum *k* value of 0.00636 min^−1^ and further attains a decrease in the *k* value as the dye concentration is enhanced. Figure 8c displays the graph between the kinetic rate constant “*k*” versus varied concentrations of MB dye (5, 10, 15, and 20 mg L^−1^).

**Figure 9:**
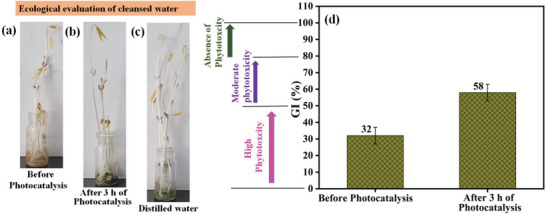
Influence of MB dye on the growth of *Vigna radiata* seeds assessed for 7 days; Assessments are performed using a) 5 mg L^−1^ MB dye, b) treated water, c) distilled water, d) Germination index evaluated after 0 and 3 h of photocatalysis.

To find the reusability, suitability, and sustainability of the treated wastewater post‐photocatalysis experiment, a basic germination index (GI) test was carried out where the seed's germination and comprehensive growth were evaluated. In each of the three vials, 10 *Vigna radiata* seeds were allocated. Every day each of these vials was added with 0.5 ml of distilled, untreated, and treated water. This test was performed for seven days duration at IIT Mandi, India having 30 °C as the environmental temperature. **Figure** **9**a–c displays the seed growth utilizing dye water prior to photocatalysis, after photocatalysis and utilizing distilled water. It could be evidenced that seed growth faced the most hindrance with not treated 5 mg L^−1^ dye while the seed growth with utilizing treated wastewater lies in the non‐toxic level.^[^ ^58^ ^]^ Edible plants require additional safety to handle their adverse implication beneficially. We would suggest using this treated water for watering the playgrounds, pavements, etc. instead of watering edible plants.^[^ ^59^ ^]^ The proper utilization of the treated water would surely lessen the load on water demand. Figure 9d displays phytotoxicity results. To evaluate the compounds correctly based on the GI values, Zucconi et. al. and Emino et. al. have provided three classifications as high phytotoxicity (*GI* < 50%), moderate phytotoxicity (50% < *GI* < 80%), and absence of phytotoxicity (*GI* > 80%).^[^ ^60^, ^61^ ^]^ As per the obtained results, untreated dye attains a high toxicity level while treated wastewater post‐photocatalysis attains a moderate level of toxicity.^[^ ^61^ ^]^ In the present case, the used treated water for the germination index attained 70% dye degradation post‐photocatalysis experiment. Thus there still lies scope to further enhance the seed germination index by using dye water with 100% purification efficiency by increasing catalytic time, catalytic load, and reducing the dye concentration.^[^ ^62^, ^63^ ^]^

**Figure 10:**
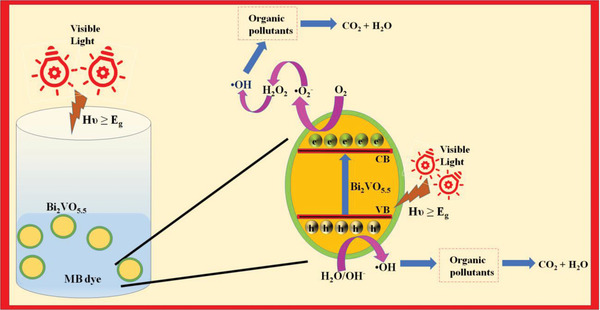
Mechanism illustration for the photocatalysis using BiV powdered sample.

**Figure** **10** displays the mechanism illustration of the photocatalytic MB dye disintegration utilizing a BiV sample. Here with the advent of visible light, there erupts electron (e^−^)‐hole (h^+^) pairs generation in the BiV phase. The holes generated undergo oxidation of the adsorbed water (H_2_O) to evolve hydroxyl radicals (OH^•^) and generated electrons interact with the adsorbed oxygen (O_2_) to evolve superoxide radicals ($O_{2}^{•-}$). In literature, these evolved species such as ($O_{2}^{•-}$) and (OH^•^) are mentioned as reactive oxidizing species (ROS) which cause MB dye to degrade into innocuous end products. Thus, dye degradation through photocatalysis occurs.^[^ ^24^, ^64^, ^65^ ^]^

## 4 Conclusion

The Bi_2_VO_5.5_ powder was produced successfully through a mechanochemical high‐energy ball milling approach at 650 °C in 5 h and its photocatalytic performance on MB dye was explored. We evidenced that ball milling enhanced the catalytic performance, as a 50% improvement in the degradation efficiency is achieved by the ball‐milled Bi_2_VO_5.5_ concerning the Not Ball‐Milled Bi_2_VO_5.5_ sample. A comparative analysis of the dye degradation performance with the use of control, ball‐milled, and not ball‐milled samples has been shown. The ball‐milled BiV sample attained 70% degradation efficiency during the photocatalysis assessment. The photocatalytic dye disintegration discerns the pseudo‐first‐order kinetic and attains the highest *k* value of 0.00636 min^−1^. As per phytotoxicity results, untreated dye attains a high toxicity level while the treated water post photocatalysis attains a moderate level of toxicity.

## Conflict of Interest

The authors declare no conflict of interest.

## Data Availability

The data that support the findings of this study are available from the corresponding author upon reasonable request.

## References

[1] W. Xie , L. Zhong , Z. Wang , F. Liang , X. Tang , C. Zou , G. Liu , Solid State Sci. 2019, 94, 1.

[2] S. Kumar , P. D. Sahare , J. Lumin. 2014, 1, 73.

[3] T. Liu , Y. G. Mao , Y. Peng , CrystEngComm 2018, 20, 2553.

[4] A. Di Paola , E. García‐López , G. Marcì , L. Palmisano , J. Hazard. Mater. 2012, 211, 3. 22169148 10.1016/j.jhazmat.2011.11.050

[5] J. Tang , Z. Zou , J. Ye , Angew. Chem. 2004, 116, 4563. 10.1002/anie.200353594 15340944

[6] N. Yang , Y. Liu , H. Wen , Z. Tang , H. Zhao , Y. Li , D. Wang , ACS Nano 2013, 7, 1504. 23350627 10.1021/nn305288z

[7] H. Chen , C. E. Nanayakkara , V. H. Grassian , Chem. Rev. 2012, 112, 5919. 23088691 10.1021/cr3002092

[8] X. Chen , L. Liu , P. Y. Yu , S. S. Mao , Science 2011, 331, 746. 21252313 10.1126/science.1200448

[9] G. A. Kallawar , D. P. Barai , B. A. Bhanvase , J Clean Prod 2021, 318, 128563.

[10] P. Chen , H. Liu , W. Cui , S. C. Lee , L. Wang , F. Dong , EcoMat 2020, 2, 12047.

[11] S. Song , Z. Xing , H. Zhao , Z. Li , W. Zhou , Green Energy Environ. 2022.

[12] J. Phanmalee , P. Intaphong , W. Kangwansupamonkon , S. Phanichphant , P. Pookmanee , Key Eng. Mater. 2017, 751, 707.

[13] P. Pookmanee , P. Intaphong , J. Phanmalee , W. Kangwansupamonkon , S. Phanichphant , Mater. Sci. Forum 2016, 872, 253.

[14] N. Kumari , S. B. Krupanidhi , K. B. R. Varma , Appl. Phys. A. 2008, 91, 693.

[15] S. M. Tauquir , M. Karnan , K. Subramani , M. Sathish , Mater. Lett. 2022, 323, 132563.

[16] K. Shantha , K. B. R. Varma , Mater. Sci. Eng., B 1999, 60, 66.

[17] Q. Luo , L. Zhang , X. Chen , O. K. Tan , K. C. Leong , RSC Adv. 2016, 6, 15796.

[18] M.‐L. Guan , D.‐K. Ma , S.‐W. Hu , Y.‐J. Chen , S.‐M. Huang , Inorg. Chem. 2011, 50, 800. 21171642 10.1021/ic101961z

[19] K. Shantha , K. B. R. Varma , J. Am. Ceram. Soc. 2000, 83, 1122.

[20] R. Venkatesan , S. Velumani , A. Kassiba , Mater. Chem. Phys. 2012, 135, 842.

[21] V.‐I. Merupo , S. Velumani , K. Ordon , N. Errien , J. Szade , A.‐H. Kassiba , CrystEngComm 2015, 17, 3366.

[22] J. M. Wang , F. Cao , X. Lv , S. Li , J. J. Cai , G. W. Qin , Mater. Sci. Forum 2016, 847, 211.

[23] W. Xie , N. Qin , B. Li , D. Bao , Ceram. Int. 2015, 41, 8433.

[24] M. Kumar , R. Vaish , S. ben Ahmed , J. Am. Ceram. Soc. 2022, 105, 2309.

[25] P. Billik , G. Plesch , Scr. Mater. 2007, 56, 979.

[26] F. Alirezazadeh , S. Sheibani , Ceram. Int. 2020, 46, 26715.

[27] L. Takacs , Prog. Mater. Sci. 2002, 47, 355.

[28] M. K. Nazemi , S. Sheibani , F. Rashchi , V. M. Gonzalez‐DelaCruz , A. Caballero , Adv. Powder Technol. 2012, 23, 833.

[29] Z. Li , Q. Zhang , L. Wu , W. Gu , Y. Liu , Adv. Powder Technol. 2019, 30, 1985.

[30] E. Dutková , M. J. Sayagués , J. Kováč , J. Kováč Jr. , Z. Bujňáková , J. Briančin , A. Zorkovská , P. Baláž , J. Ficeriová , Mater. Lett. 2016, 173, 182.

[31] K. Kucio , B. Charmas , S. Pasieczna‐Patkowska , M. Zięzio , Appl. Nanosci. 2020, 10, 4733.

[32] A. Ebrahimi‐Purkani , S. F. Kashani‐Bozorg , J. Alloys Compd. 2008, 456, 211.

[33] C. C. Koch , Y. S. Cho , Nanostruct. Mater. 1992, 1, 207.

[34] D. Zhang , C. Su , S. Yao , H. Li , X. Pu , Y. Geng , J Phys Chem Solids 2020, 147, 109630.

[35] Z. Shao , X. Meng , H. Lai , D. Zhang , X. Pu , C. Su , H. Li , X. Ren , Y. Geng , Chin. J. Catal. 2021, 42, 439.

[36] G. Nabi , N. Malik , W. Raza , Inorg. Chem. Commun. 2020, 119, 108050.

[37] S. S. R. Albeladi , M. A. Malik , S. A. Al‐thabaiti , J. Mater. Res. Technol. 2020, 9, 10031.

[38] N. A. Wójcik , M. Prześniak‐Welenc , P. Kupracz , J. Karczewski , M. Gazda , R. J. Barczyński , Phys. Status Solidi B 2017, 254, 1700093.

[39] S. Kumar , P. D. Sahare , Nano 2013, 8, 1350007.

[40] S. J. Patwe , A. Patra , R. Dey , A. Roy , R. M. Kadam , S. N. Achary , A. K. Tyagi , J. Am. Ceram. Soc. 2013, 96, 3448.

[41] K. Anwar , F. K. Naqvi , S. Beg , Phase Transitions 2022, 95, 64.

[42] C. Lv , G. Chen , X. Zhou , C. Zhang , Z. Wang , B. Zhao , D. Li , ACS Appl Mater Interfaces 2017, 9, 23748. 28653534 10.1021/acsami.7b05302

[43] Z. Liu , J. Niu , P. Feng , Y. Sui , Y. Zhu , RSC Adv. 2014, 4, 43399.

[44] N. Daelman , F. S. Hegner , M. Rellán‐Piñeiro , M. Capdevila‐Cortada , R. García‐Muelas , N. López , J. Chem. Phys. 2020, 152, 050901. 32035446 10.1063/1.5138484

[45] C.‐M. Fan , Y. Peng , Q. Zhu , L. Lin , R.‐X. Wang , A.‐W. Xu , J. Phys. Chem. 2013, 117, 24157.

[46] J. Xia , W. Liu , Y. Teng , Q. S. Wang , L. Zhao , M. M. Ruan , RSC Adv. 2015, 5, 12015.

[47] C. Chuaicham , K. Sekar , Y. Xiong , V. Balakumar , Y. Mittraphab , K. Shimizu , B. Ohtani , I. Dabo , K. Sasaki , Chem. Eng. J. 2021, 425, 130502.

[48] F. Yu , F. Gong , Q. Yang , Y. Wang , Diamond Relat. Mater. 2022, 125, 109004.

[49] E. Prabakaran , T. Velempini , M. Molefe , K. Pillay , J. Mater. Res. Technol. 2021, 15, 6340.

[50] Z. Li , M. Chen , Z. Ai , L. Wu , Q. Zhang , Appl. Clay Sci. 2018, 163, 265.

[51] Z. Li , Q. Zhang , X. Liu , M. Chen , L. Wu , Z. Ai , Appl. Surf. Sci. 2018, 452, 123.

[52] S. Chen , Y. Hu , X. Jiang , S. Meng , X. Fu , Mater. Chem. Phys. 2015, 149, 512.

[53] M. Jalalah , M. Faisal , H. Bouzid , A. A. Ismail , S. A. Al‐Sayari , Mater. Res. Bull. 2013, 48, 3351.

[54] G. He , J. Ding , J. Zhang , Q. Hao , H. Chen , Ind. Eng. Chem. Res. 2015, 54, 2862.

[55] E. Mendoza‐Mendoza , A. G. Nuñez‐Briones , R. Leyva‐Ramos , R. D. Peralta‐Rodríguez , L. A. García‐Cerda , E. D. Barriga‐Castro , R. Ocampo‐Pérez , J. Rodríguez‐Hernández , Ceram. Int. 2019, 45, 6268.

[56] R. P. Singh , P. S. Khagar , A. K. Mourya , S. K. Warkhade , S. P. Zodape , U. R. Pratap , A. V. Wankhade , Mater. Sci. Semicond. Process. 2022, 143, 106526.

[57] S. S. Sharma , S. Palaty , A. K. John , Int. J. Environ. Sci. Technol. 2021, 18, 2619.

[58] A. Priac , P.‐M. Badot , G. Crini , C. R. Biol. 2017, 340, 188. 28256413 10.1016/j.crvi.2017.01.002

[59] A. Singh , A. Bhati , P. Khare , K. M. Tripathi , S. K. Sonkar , Sci. Rep. 2019, 9, 2522. 30792461 10.1038/s41598-019-38717-1 PMC6384933

[60] E. R. Emino , P. R. Warman , Compost Sci. Util. 2004, 12, 342.

[61] F. Zucconi , A. Monaco , M. Forte , in Composting of Agricultural and other Wastes, (Ed: J. K. R. Gasser ), Elsevier, New York 1985, pp. 73–86.

[62] S. Tu , Y. Guo , Y. Zhang , C. Hu , T. Zhang , T. Ma , H. Huang , Adv. Funct. Mater. 2020, 30, 2005158.

[63] A. Kumar , G. Pandey , Mater. Sci. Eng. Int. J. 2017, 1, 106.

[64] Y. Zhao , X. Liu , S. Gu , J. Liu , RSC Adv. 2021, 11, 9746. 35423437 10.1039/d1ra00055a PMC8695501

[65] X. Kong , X. Liu , Y. Zheng , P. K. Chu , Y. Zhang , S. Wu , Mater. Sci. Eng., R 2021, 145, 100610.

